# Age-related differences in lower limb muscle activation patterns and balance control strategies while walking over a compliant surface

**DOI:** 10.1038/s41598-023-43728-0

**Published:** 2023-10-02

**Authors:** Woohyoung Jeon, Ahmed Ramadan, Jill Whitall, Nesreen Alissa, Kelly Westlake

**Affiliations:** 1 Department of Health and Kinesiology, University of Texas at Tyler, Tyler, TX USA; 2 Department of Biomedical Engineering, University of Minnesota Twin Cities, Minneapolis, MN USA; 3 Department of Physical Therapy and Rehabilitation Science, University of Maryland School of Medicine, Baltimore, MD USA

**Keywords:** Medical research, Risk factors

## Abstract

Substantial evidence demonstrates that falls in older adults are leading causes of fatal and non-fatal injuries and lead to negative impacts on the quality of life in the aging population. Most falls in older fallers result from unrecoverable limb collapse during falling momentum control in the single limb support (SLS) phase. To understand why older adults are more likely to fall than younger adults, we investigated age-related differences in knee extensor eccentric control, lower limb muscle activation patterns, and their relation to balance control. Ten older and ten younger healthy adults were compared during balance control while walking on a compliant surface. There was a positive correlation between knee extensor eccentric work in the perturbed leg and the swinging leg’s speed and margin of stability. In comparison to younger adults, older adults demonstrated (1) less eccentric work, reduced eccentric electromyography burst duration in the perturbed leg, (2) higher postural sway during SLS, and (3) impaired swinging leg balance control. The group-specific muscle synergy showed that older adults had a prominent ankle muscle activation, while younger adults exhibited a more prominent hip muscle activation. These findings provide insight into targeted balance rehabilitation directions to improve postural stability and reduce falls in older adults.

## Introduction

A decrease in the ability to adapt locomotor control to varied surface properties is part of normal aging^1^. While walking over multi-terrain surfaces, such as solid, compliant, rocky, irregular, slippery, and uneven surfaces, older adults demonstrate decreased gait speed, reduced step length, and increased variability in medial–lateral center of mass (CoM) acceleration and trunk roll^1,2^. Previous studies over firm, level ground surfaces have found that age-related impairment in neuromuscular control and force-generating capacity in the lower limbs lead to altered muscle activation patterns during walking balance recovery^3–5^. For example, neuromuscular control tends to become more simplistic with age, leading to reduced variability and efficiency of muscle synergy options^3^. In addition, age-related reductions in lower limb force generation leads to altered muscle activation patterns while walking. During the push-off phase of gait, older adults demonstrate greater magnitude of ankle plantar flexor electromyography (EMG) activation compared to younger adults despite having no age-related difference in plantar flexion torque^6^. This relatively inefficient control of ankle joint forces leads to altered muscle activation patterns, which redistributes push-off force generation to more proximal muscles at the knee and hip joints^7^. Older adults also demonstrate changes in movement strategies with ineffective inter-joint coordination, such as greater agonist and antagonist coactivation at the ankle and knee joints during downhill walking^4^, and increased joint kinematic variability at the ankle, knee, and hip joints during walking with lateral balance perturbations^8^. Nevertheless, although alterations in neuromuscular movement strategies and force-generation capacity have been demonstrated while walking over level surfaces, less is known regarding how these control mechanisms are affected by age while walking over irregular or compliant surfaces.

Surfaces such as muddy ground, grass, sandy soil, or plush carpet place greater demands on reactive locomotor strategies that include critical online task related real-time sensory feedback and integration^9^. During unexpected slips, for example, a key response mechanism during the single limb support (SLS) phase of gait is eccentric work of the quadriceps to control CoM displacement^10^. Without adequate eccentric control, either a fall or postural instability in the vertical, anterior–posterior (A-P) and/or mediolateral (M-L) directions will ensue^11^. Previously identified age-related increases in force-generating errors and larger force variability when producing knee extensor eccentric control^12^ are important mechanistic considerations that may underlie limb collapse and instability during SLS in older adults. These findings also demonstrate that when standing on a compliant surface, older adults exhibit greater postural sway and more rigid postural behavior compared to younger adults. Therefore, an investigation of age-related changes in neuromuscular eccentric control characteristics at the knee joint and differences in lower limb muscle coordination patterns to secure balance during SLS can reveal key factors underlying increased fall risk in older adults, especially after an encounter with unpredictable ground surface compliance challenges while walking.

The purpose of this study was to investigate (1) age-related differences in the characteristics of knee extensor eccentric control and muscle coordination patterns of the perturbed leg and swinging (trailing) leg protective step balance control while walking on a compliant (foam) surface and (2) the relationship between perturbed leg eccentric control and postural stability (postural sway) during the SLS phase and swinging leg protective stepping response. The results of this investigation will provide insights into novel directions for lower limb strengthening exercises to improve dynamic balance control and reduce fall risk in an aging population.

## Methods

### Participants

Ten healthy younger adults (24 ± 3 years; 6 females, 4 males) and ten healthy older adults (77 ± 8 years; 5 females, 5 males) participated in this study. Physical activity level (the number of days and hours spent walking and doing physical activities per week) was measured using International Physical Activity Questionnaire^13^. Participants were included in this study if they (1) were able to walk 10 m without assistive devices and (2) had a “moderate” or higher physical activity level, which meets the following criteria:

(a) three or more days of vigorous-intensity activity of at least 20 min per day OR (b) five or more days of moderate-intensity activity and/or walking of at least 30 min per day OR c) five or more days of any combination of walking, moderate-intensity or vigorous intensity activities achieving a minimum total physical activity of at least 600 min/week. Participants’ characteristics are listed in Table 1.

**Table 1:** Anthropometrics and spatiotemporal gait characteristics of study participants across age groups.

Characteristics	Older adults (n = 10)	Younger adults (n = 10)	*P* value
Anthropometric			
Age (years)	77 ± 8	24 ± 3	
Sex (female/male)	5 / 5	6 / 4	
Height (cm)	170.8 ± 9.36	170.0 ± 8.77	0.85
Weight (kg)	75.33 ± 13.62	73.74 ± 21.55	0.84
BMI (kg/m^2^)	24.02 ± 0.86	22.44 ± 3.08	0.13
Gait			
Gait Speed (cm/s)	112.23 ± 21.23	121.14 ± 11.30	0.25
Gait Initiation step length (cm)	49.33 ± 9.33	55.90 ± 5.40	0.07
Step length (cm)	61.24 ± 7.96	68.92 ± 6.10	0.03*
Single stance duration (s)	0.43 ± 0.10	0.41 ± 0.02	0.49
H–H base of support (cm)	9.29 ± 2.61	11.40 ± 3.17	0.12

Values are presented as mean ± standard deviation. Single stance duration is equal to the swing time of the opposite foot. H–H (heel-to-heel) base of support is “base width”, which is the vertical distance from heel center of one footprint to the line of progression formed by two footprints of the opposite foot. * represents a significant difference between the two groups.

Participants were excluded from this study if they had (1) deficits or disorders that could affect balance control; (2) history of dizziness and imbalance; (3) history of neurological (e.g., Parkinson’s disease, Alzheimer’s disease, stroke, visual and/or vestibular impairment), musculoskeletal, or any other systemic disorders; and (4) Body Mass Index (BMI) within the overweight and obesity range (BMI is higher than 25 kg/m^2^). All procedures were approved by the Institutional Review Board at the University of Maryland (protocol code: HP-00093655 and dated 1/21/2021) and were in accordance with the Helsinki Declaration of 1975. All participants provided written informed consent prior to study participation.

### Data collection

A wireless EMG System (TeleMyo, Noraxon, Scottsdale, AZ) was used for the acquisition of muscle activity signals. Noraxon adhesive pre-gelled Ag/AgCl surface EMG electrodes (40 × 21 mm, inter-electrode distance: 20 mm) were placed bilaterally on the tibialis anterior (TA), medial gastrocnemius (mGas), rectus femoris (RF), biceps femoris (BF), gluteus medius (Gmed), and erector spinae (ES). The positioning of the electrodes was in accordance with SENIAM guidelines^14^. To normalize EMG amplitude for each muscle, participants performed a segment weight dynamic movement (SWDM) for each joint (ankle, knee, and hip) before collecting data^15^. Thirty-nine reflective markers were placed on the body according to the standard Plug-In-Gait full-body model (Vicon Nexus 2.12, VICON Motion Systems Ltd, UK). A 10-camera motion capture system (VICON Motion Systems Ltd, UK) was used to record body kinematics.

Walking on a compliant (foam) surface, balance challenge was measured at heel strike of gait initiation to standardize the timing of the perturbation and to normalize kinematic responses to the same time period of the gait cycle. Before the start of testing, spatiotemporal gait data (usual walking speed and step length) during unperturbed gait were obtained from 3 trials on the GaitRite mat (Protokinetics, Havertown, PA). Step length was used to estimate the starting position for each participant to ensure that heel strike of the leading (perturbed) leg occurred at a center location on the compliant (foam) surface platform. Because we were interested in the naïve response to ground walking balance challenge, we only measured the first exposure to balance challenge on the compliant (foam) surface, which requires active postural balance control during SLS to counteract the unpredictable proprioceptive sensory inputs caused by a foam surface. It should be noted that the participants could anticipate a compliant surface since it was not hidden from them, yet the surface properties (e.g., stiffness and damping) were unknown to the participants.

**Figure 1:**
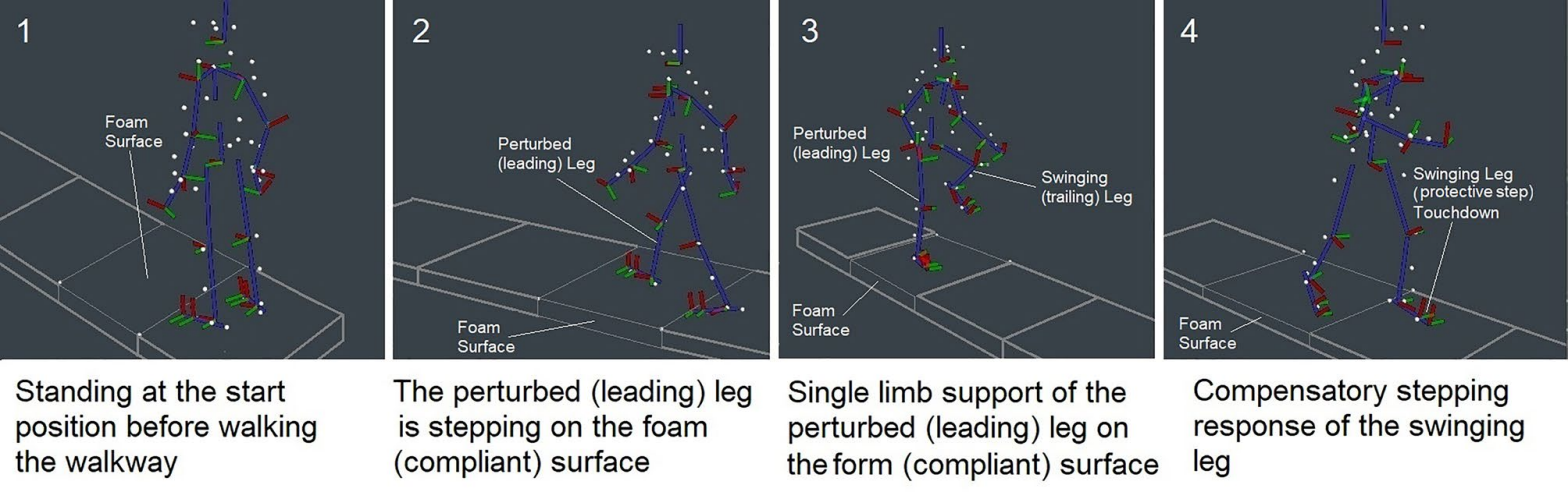
Experimental setup for walking on a compliant surface. A participant was standing at the start position (1), anticipating of a compliant surface. A compliant surface drop occurred at heel strike of the leading leg (2) during gait initiation. Eccentric control of the knee and body’s postural sway were measured during the single limb support phase (3) of the leading (perturbed) leg stepping. The trailing (swinging) leg stepped onto the firm surface of the platform (4) and margin of stability of the trailing leg (protective step) was measured.

During testing, participants wore a safety harness linked with a rope to an overhead pulley system with little resistance. The length of the rope was adjusted to participants’ height so that they could walk freely. Participants were asked to walk at their normal pace along a 30’ (9.14 m) walkway (U.S. Provisional Pat. Ser. No. 62/949,184). The walkway was modular and the first 3’ module was located before a compliant (foam) surface module (Fig. 1). Instructions to participants were as follows: “Walk to the end of the walkway at your normal walking pace. Your balance may or may not be challenged. If your balance is disturbed, react naturally, and keep walking to the end of the walkway”.

### Data processing

EMG data were sampled at 1,500 Hz and kinematic data was sampled at 150 Hz. EMG, kinematics, and kinetics data were analyzed in MATLAB 2022b (MathWorks Inc., Natick, MA, USA).

#### EMG

##### Surface EMG signal processing

The raw surface EMG (sEMG) data collected during responses to perturbations underwent the following processing steps:

1. Pre-processing of sEMG signal: Any DC offset was first eliminated using the “detrend” function in MATLAB. Next, a median filter was applied to the signal to remove noise^16^, followed by the application of a 20—450 Hz band-pass filter to extract the frequency range where muscular energy is concentrated^17,18^.
2. sEMG rectification and linear envelope: sEMG signal values below zero were converted to positive values of the same amplitude to create a full-wave rectified sEMG signal^19^. To obtain sEMG envelopes, a 2nd order Butterworth low pass filter with a 20 Hz cutoff frequency was applied as a digital smoothing^20^.

##### Knee extensor eccentric control

**Figure 2:**
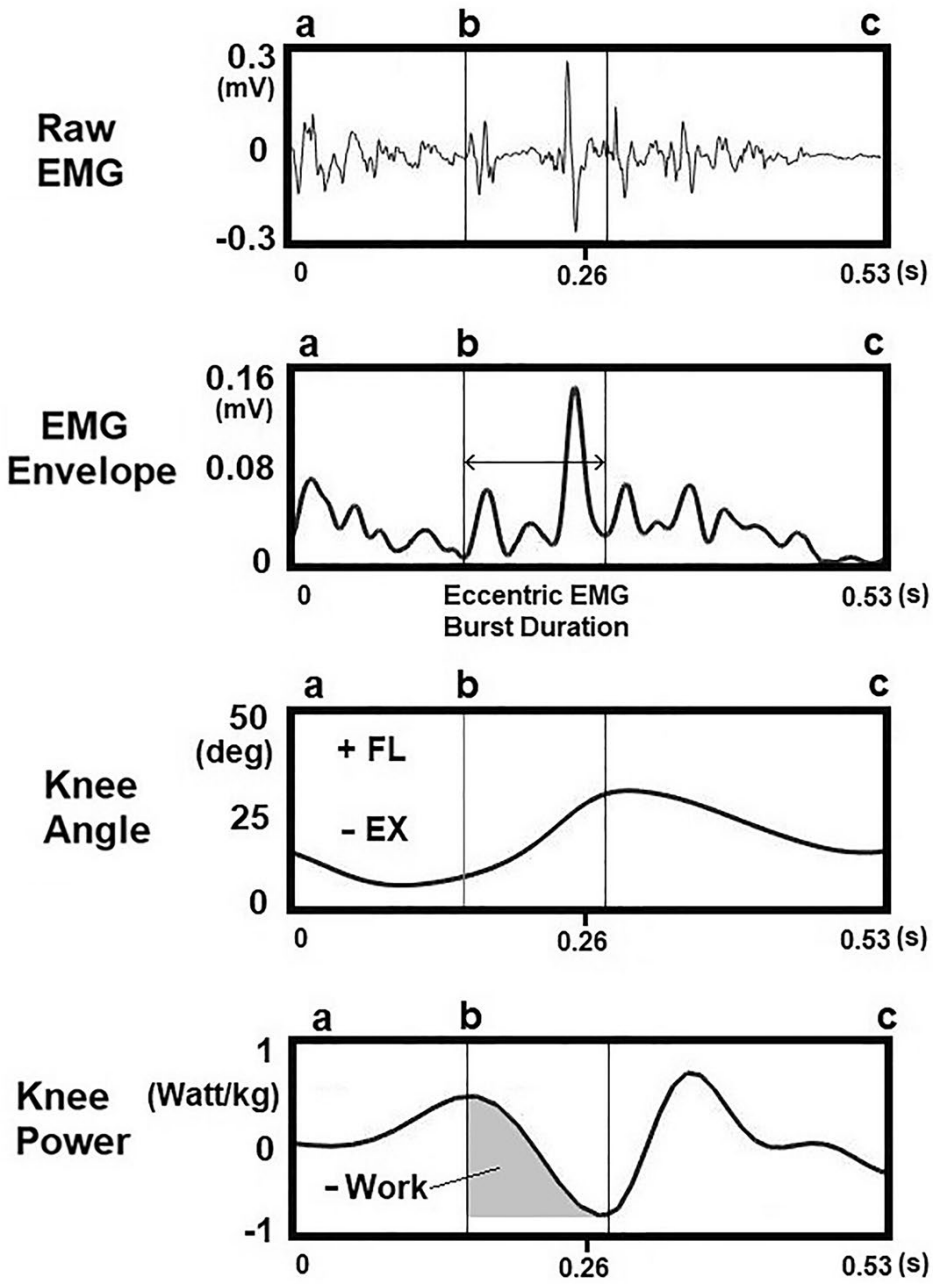
A representative figure depicts calculation of EMG burst duration (duration between the vertical lines) and negative eccentric work (-work) of the perturbed leg during balance control in the single limb support (SLS) phase. Eccentric work was calculated by the integration of the power-time curve where negative work indicates an eccentric RF contraction (the shaded area under curve). Knee angle (-: extension, + : flexion). (**a**) Surface drop start, (**b**) Start of perturbed leg eccentric contraction, (**c**) Swinging (trailing) leg touch down the platform.

EMG burst duration onset of the RF in the perturbed leg was defined as the time of minimal EMG activity after the instant of knee angle transition from extension (concentric) to flexion (eccentric) following surface drop perturbation (stepping on the compliant surface) start. The end of EMG burst duration was determined as the time of minimum EMG value at the instant of knee angle transition from flexion (eccentric) to extension (concentric) following the highest EMG peak of the eccentric contraction (Fig. 2). EMG peak latency was defined as the period from the instant when surface drop perturbation starts (perturbed leg stepping on the compliant surface) until the eccentric EMG amplitude reached its peak.

##### Muscle synergy

To characterize the muscle activation patterns for balance recovery strategies, we performed muscle synergy analysis using non-negative matrix factorization (NNMF). Prior to NNMF decomposition for muscle synergy extraction, we employed a moving root mean square (RMS) with a window length of 100 samples to create the sEMG envelopes^21^. Since we are interested in naïve response during the first exposure (a single trial) to the balance challenge on the compliant (foam) surface, the synergies were extracted from this single trial. An EMG matrix was constructed where the rows are the perturbed leg muscles, while the columns are the sampled data during the SLS phase.

*Extraction of muscle synergy*: Nonnegative matrix factorization (NNMF), using a multiplicative iterative algorithm, extracted muscle synergies (muscle-weighting and temporal synergy activation) from the EMG matrix^22,23^ NNMF decomposes the EMG signals of a specific muscle activation pattern within a given time period into two distinct components:

1. W: This vector specifies the spatial pattern of the relative activation level of each muscle in the muscle synergy. Each muscle’s contribution is relatively weighted within this spatial structure.
2. C: This scaling coefficient represents the temporal synergy activation. The spatial components are multiplied by a scaling (synergy recruitment) coefficient C. This transformation can be expressed as:
  1 $${\text{EMG}}_{{0 \, ({\text{m}} \times {\text{t}})}} = {\text{W}}_{{({\text{m}} \times {\text{n}})}} \cdot {\text{ C}}_{{({\text{n}} \times {\text{t}})}} + {\text{e}} = {\text{EMGr}}_{{({\text{m}} \times {\text{t}})}} {\text{e}},$$

where m = the number of muscles, t = the number of time points, n = the number of muscle synergies, e = residual error, and EMGr = reconstructed EMG matrix.

The spatial components (W) are fixed time-invariant patterns, whereas the temporal synergy activation coefficient (C) varies over time^24^. Therefore, the coefficient (C) specifies how the coordinated muscle activation pattern is modulated over time during the targeted movement period (the supplementary material displays the two components of each muscle synergy extracted by NNMF: (1) temporal synergy activation and (2) spatial structure of relative muscle activation levels).

To evaluate the similarity between EMG_0_ and EMGr, variability accounted for (VAF) was calculated according to the following equation:

2 $${\text{VAF}} = \left( {1 - \frac{{\left( {{\text{EMG}}_{0} \user2{ }{-}{\text{ EMG}}_{r} } \right)^{2} }}{{\left( {{\text{EMG}}_{0} - {\text{mean}}\left( {{\text{EMG}}_{0} } \right)} \right)^{2} }}} \right) \times 100\%$$

To determine the optimal number of synergies, we repeated the optimization to extract k (from 1 to the number of EMG sensors) synergies and the associated VAF. Then the smallest k with VAF > 90% was selected^25^.

k-means clustering algorithm (from MATLAB R2022b) categorized the similar groups of muscle synergies across all participants. Then, intra-class correlation coefficient (ICC) was used to examine the internal consistency of all muscle synergies. Common muscle synergies between older and younger adult groups with ICC over 0.75, indicating good reliability, were categorized in the same cluster. To determine age-specific synergies, the ICC value of muscle synergies over 0.9, indicating excellent reliability, were categorized in the same cluster^26^.

#### Kinematic and kinetic data

The body’s center of mass (CoM) trajectory, knee joint angular displacement and power were calculated using Vicon Nexus 2.12 Software (Vicon, Oxford Metrics, UK). Kinematic and kinetic data were Butterworth low-pass filtered at 6 Hz and 25 Hz, respectively^27^.

*Kinematics*: To measure protective stepping stability, we used the margins of stability (MoS). MoS is the minimum distance from extrapolated CoM to the posterior boundary of the base of support (BoS) in the A-P direction. The extrapolated CoM was quantified as x + v/ω_0_, where x and v are the CoM position and instantaneous CoM velocity respectively, and ω_0_ is angular frequency = $\sqrt{g/l}$, ‘g’ is the gravity, ‘l’ is the length between the CoM and foot. The kinematic data during walking balance control were normalized to the unperturbed walking kinematic data (value in perturbed trial / value in unperturbed walking) × 100%). The toe marker trajectory in the A-P and vertical direction was used to calculate maximal velocity of the swinging (trailing) leg protective step. Postural sway (body oscillation) during the eccentric work phase of the perturbed leg was calculated to examine postural stability. To quantify postural sway, standard deviation (SD) of CoM acceleration (SDCoMAccel) in the A-P, M-L, and vertical directions were computed.

*Kinetics*: Work at the ankle, knee, and hip joints was calculated by the integration of the power-time curve. Knee joint eccentric work in the A-P direction represented the negative work (Fig. 2) resulting from eccentric muscle contraction, facilitating power absorption at the knee joint during the perturbed leg knee flexion^28^. In the M-L direction, ankle joint inversion work aimed to laterally shift center of pressure, ensuring the CoM remains within the base of support, while hip abduction work aimed to shift the CoM towards the base of support’s center. All work data was normalized to body weight (J/kg).

### Statistical analysis

A statistical software (IBM SPSS Statistics 25; Chicago, IL, USA) was used for all statistical analysis with an established a-priori alpha level of 0.05. For the justification of our sample size, an a priori power analysis was conducted using G*Power. Effect size (Cohen’s *d*) was calculated based on previous studies^28–30^, resulting in an effect size of 1.35. In G*Power, a T-test was selected as the test family to assess mean differences between two groups. This was a two-tailed test with 80% power (1 − β, the Type II error rate) at the 0.05 alpha level (Type I error rate). Shapiro–Wilk test was performed to test the normality. All EMG, kinetic, kinematic data were normally distributed.

A one-way multivariate analysis of variance (MANOVA) was used to determine whether there were any differences between age groups (old and young) on (1) knee extensor eccentric control; knee extensor eccentric work and RF EMG activity (eccentric EMG burst duration, normalized EMG amplitude (% EMG SWDM), and EMG peak latency from the instant when perturbed leg stepping on the compliant (foam) surface) during SLS, (2) postural sway (SDCoMAccel), (3) MoS of first compensatory step of the trailing leg touchdown following SLS on the compliant (foam) surface and (4) maximum velocity of swinging leg. The Bonferroni test was used for post hoc analyses, pairwise comparisons between two groups, where indicated. Pearson’s correlation (r) was used to examine the correlation between eccentric work in the perturbed leg and swinging leg balance responses: (1) MoS and (2) Maximum velocity of swinging leg for compensatory step).

## Results

All data are presented as mean ± standard deviation (SD). All participants demonstrated a swinging (trailing) leg protective stepping response in the forward direction without a fall. As we obtained age-specific synergies from each age group, our analysis focused on understanding the composition of age-specific muscle synergies (the spatial structure of relative muscle activation levels during a balance response), rather than assessing the relative contributions of each synergy to each muscle. We investigated the relative contributions of individual muscles to age-specific synergies to identify age-specific postural control strategies in terms of muscle activation patterns for regaining postural stability after ground balance challenges.

### Perturbed leg knee extensor eccentric control during SLS

**Figure 3:**
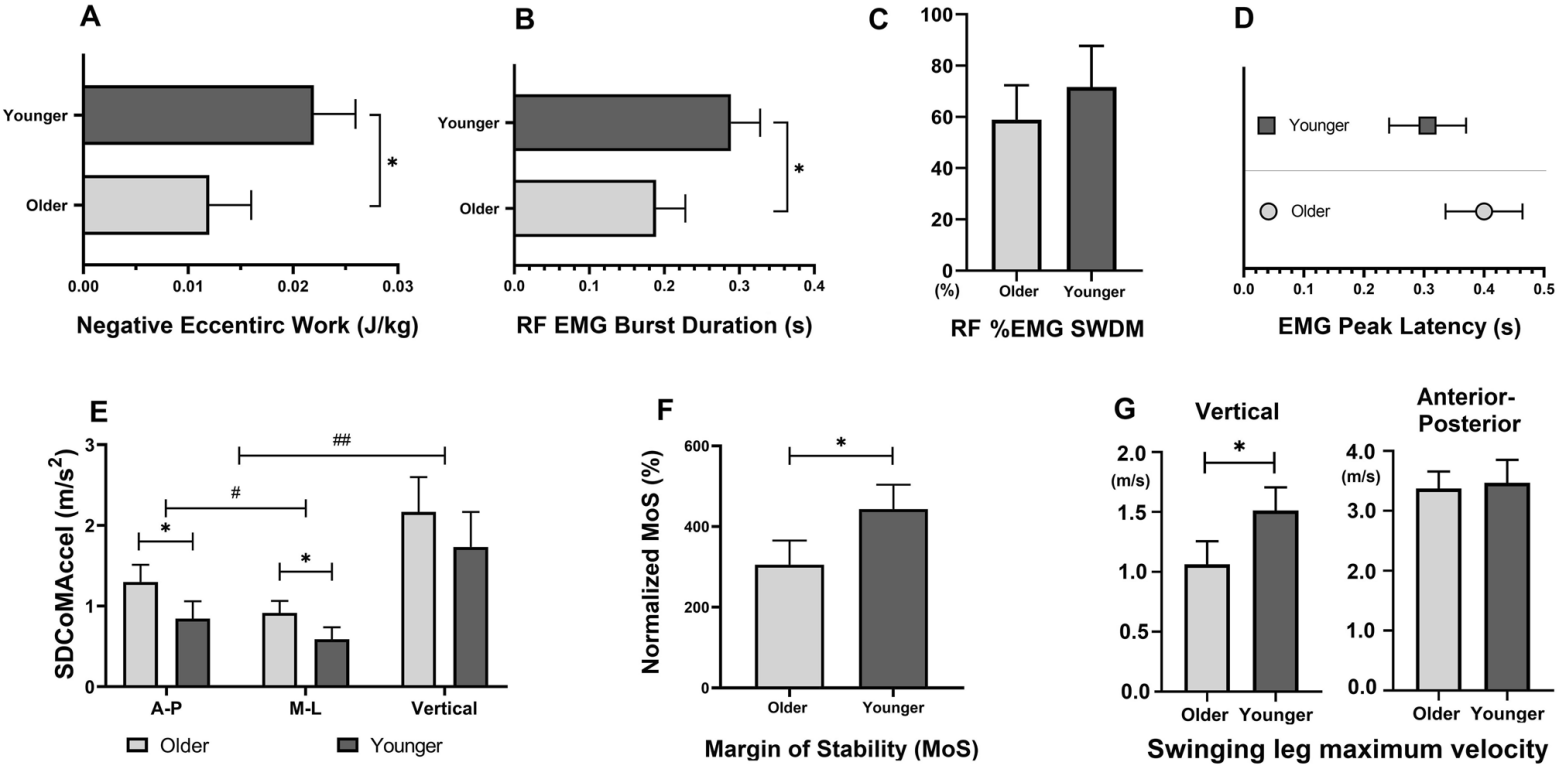
Older versus younger (light versus dark grey) biomechanical measures: (**A**) Negative Eccentric Work (J/kg). (**B**) Rectus femoris eccentric contraction EMG burst duration (s). (**C**) Rectus femoris %EMG segment weight dynamic movement (SWDM). (**D**) Rectus femoris eccentric contraction EMG peak latency (s) (**E**) Postural sway (SD of CoM acceleration) during SLS in the A-P, M-L, and vertical directions. (**F**) Margin of Stability (normalized to normal gait, %) of first compensatory step touchdown following perturbations. (**G**) Maximum vertical velocity of swinging leg for compensatory step. * represent a statistically significant difference between older and younger adults (*p* < 0.05). The error bars display the standard error. # represent a statistically significant difference between the directions (*p* < 0.05).

Compared to younger adults, older adults demonstrated reduced eccentric knee extensor work (older: 0.011 ± 0.008 J/kg, younger: 0.022 ± 0.010 J/kg, *p* = 0.027, Fig. 3A) and RF EMG burst duration (older: 0.189 ± 0.029 s, younger: 0.289 ± 0.120 s, *p* = 0.021, Fig. 3B). There was no difference in % EMG SWDM (older: 58.97 ± 13.41%, younger: 71.67 ± 16.05%, *p* = 0.07, Fig. 3C) and EMG peak latency (older: 0.400 ± 0.116 s, younger: 0.306 ± 0.163 s, *p* = 0.158, Fig. 3D).

### Posutral sway during SLS

Compared to younger adults, older adults demonstrated greater postural sway (SDCoMAccel) in the A-P(older: 1.298 ± 0.185 m/s^2^, younger: 0.084 ± 0.010 m/s^2^, *p* = 0.047) and M-L directions (older: 0.914 ± 0.141 m/s^2^, younger: 0.587 ± 0.047 m/s^2^, *p* = 0.041, Fig. 3E), whereas there was no difference in the vertical direction (older: 2.165 ± 0.274 m/s^2^, younger: 1.733 ± 0.204 m/s^2^, *p* = 0.222).

### MoS of first compensatory step and swining leg maximum velocity

Compared to younger adults, older adults demonstrated reduced MoS (older: 305.43 ± 30.98%, younger: 443.72 ± 51.39%, *p* = 0.033, Fig. 3F) and decreased swinging leg maximum velocity in the vertical direction (older: 1.062 ± 0.052 m/s, younger: 1.511 ± 0.185 m/s, *p* = 0.041, Fig. 3G), whereas there was no difference in the A-P direction (older: 3.308 ± 0.255 m/s, younger: 3.488 ± 0.303 m/s, *p* = 0.655).

### Work for postural control at the ankle and hip joints

**Figure 4:**
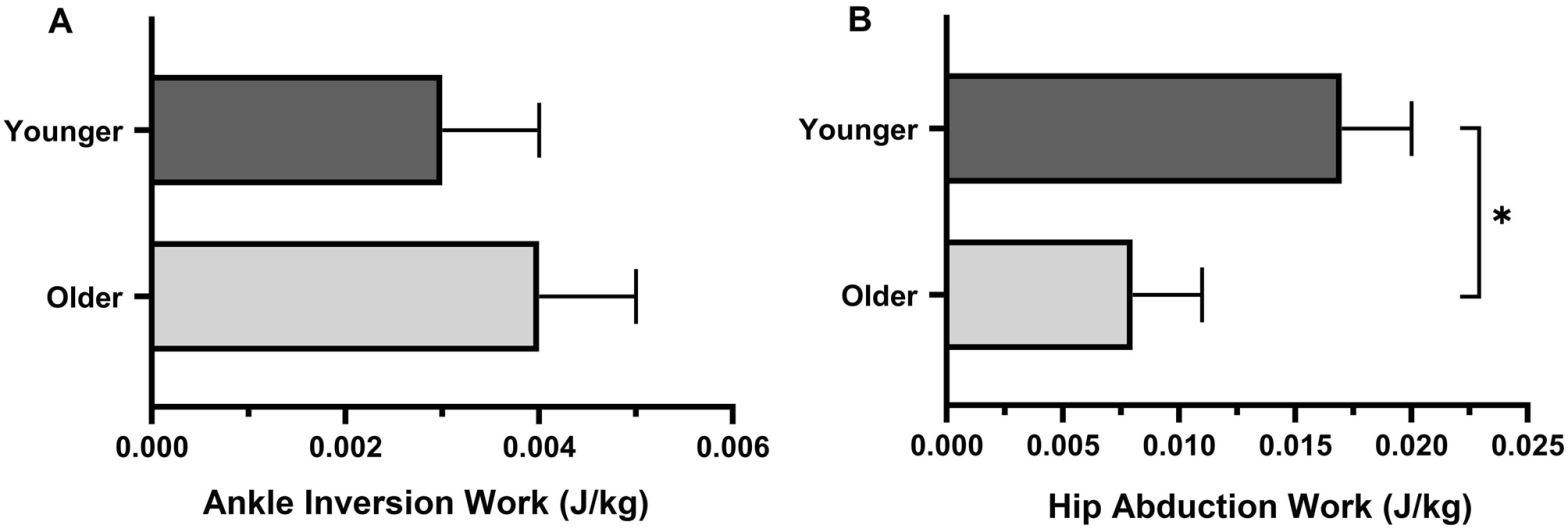
Differences in work (J/kg) for postural control between older and younger adults at the ankle and hip joints during the single limb support phase. (**A**) Ankle inversion work aimed to laterally shift center of pressure, ensuring the CoM remains within the base of support. (**B**) Hip abduction work aimed to shift the CoM towards the base of support’s center.

Compared to younger adults, older adults showed a tendency to have greater ankle joint inversion work (older: 0.004 ± 0.001 J/kg, younger: 0.003 ± 0.001 J/kg, *p* = 0.56, Fig. 4A). However, there was no statistically significant difference between the two groups. At the hip joint level, younger adults demonstrated increased hip abduction work compared to older adults (older: 0.008 ± 0.003 J/kg, younger: 0.017 ± 0.003 J/kg, *p* = 0.035, Fig. 4B).

### Correlation between eccentric work in the perturbed leg and swinging (trailing) leg balance responses

**Figure 5:**
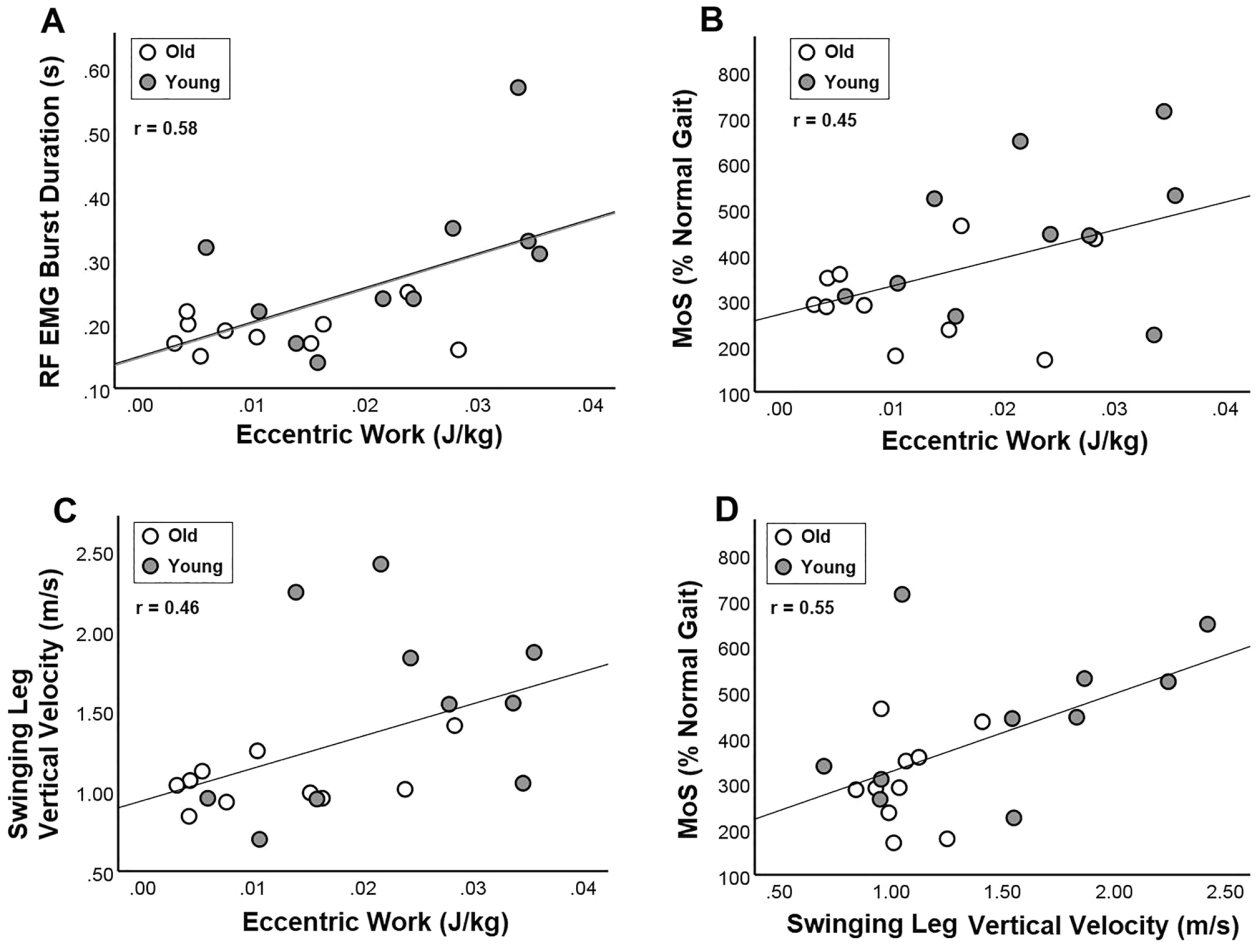
(**A**) Correlation between eccentric work of the perturbed leg and RF EMG burst duration (s) (r = 0.58, *p* = 0.01). (**B**) Correlation between eccentric work of the perturbed leg and margin of stability of first compensatory step touchdown (r = 0.45, *p* = 0.04). (**C**) Correlation between rectus femoris eccentric EMG burst duration and maximum velocity of swinging leg for compensatory step touchdown (r = 0.46, *p* = 0.04). (**D**) Correlation between maximum velocity of swinging leg and margin of stability (r = 0.54, *p* = 0.01).

Across all participants, there were positive correlations between eccentric work in the perturbed leg and (1) RF EMG burst duration (Fig. 5A), (2) MoS of compensatory step (r = 0.45, *p* = 0.04, Fig. 5B), and (3) swinging leg maximum vertical velocity (r = 0.46, *p* = 0.04), Fig. 5C). Additionally, a positive relationship between swinging leg maximum vertical velocity and MoS (r = 0.54, *p* = 0.01, Fig. 5D) was observed.

### Muscle synergy

**Figure 6:**
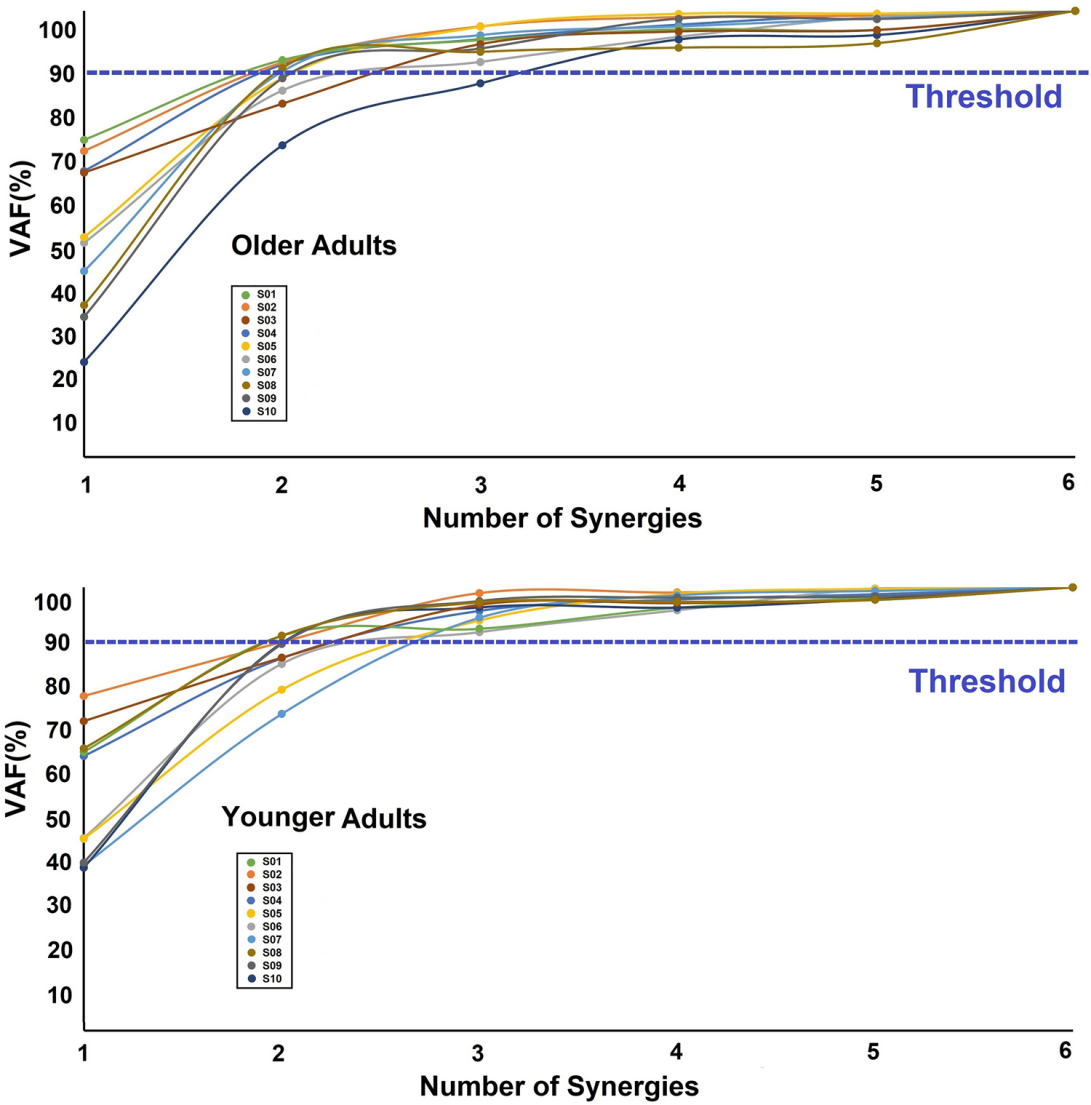
The number of muscle synergies that meet the condition of VAF (Variability Accounted For) ≥ 90% for each subject is shown. The dashed line indicates the VAF threshold. Abbreviations: O (older adults), Y (younger adults).

In the early and middle phases of SLS, the number of synergies extracted was 3.05 ± 0.51 and there was no difference between two groups (older adults 3.10 ± 0.56, younger adults 3.00 ± 0.47, *p* = 0.40, Fig. 6).

**Figure 7:**
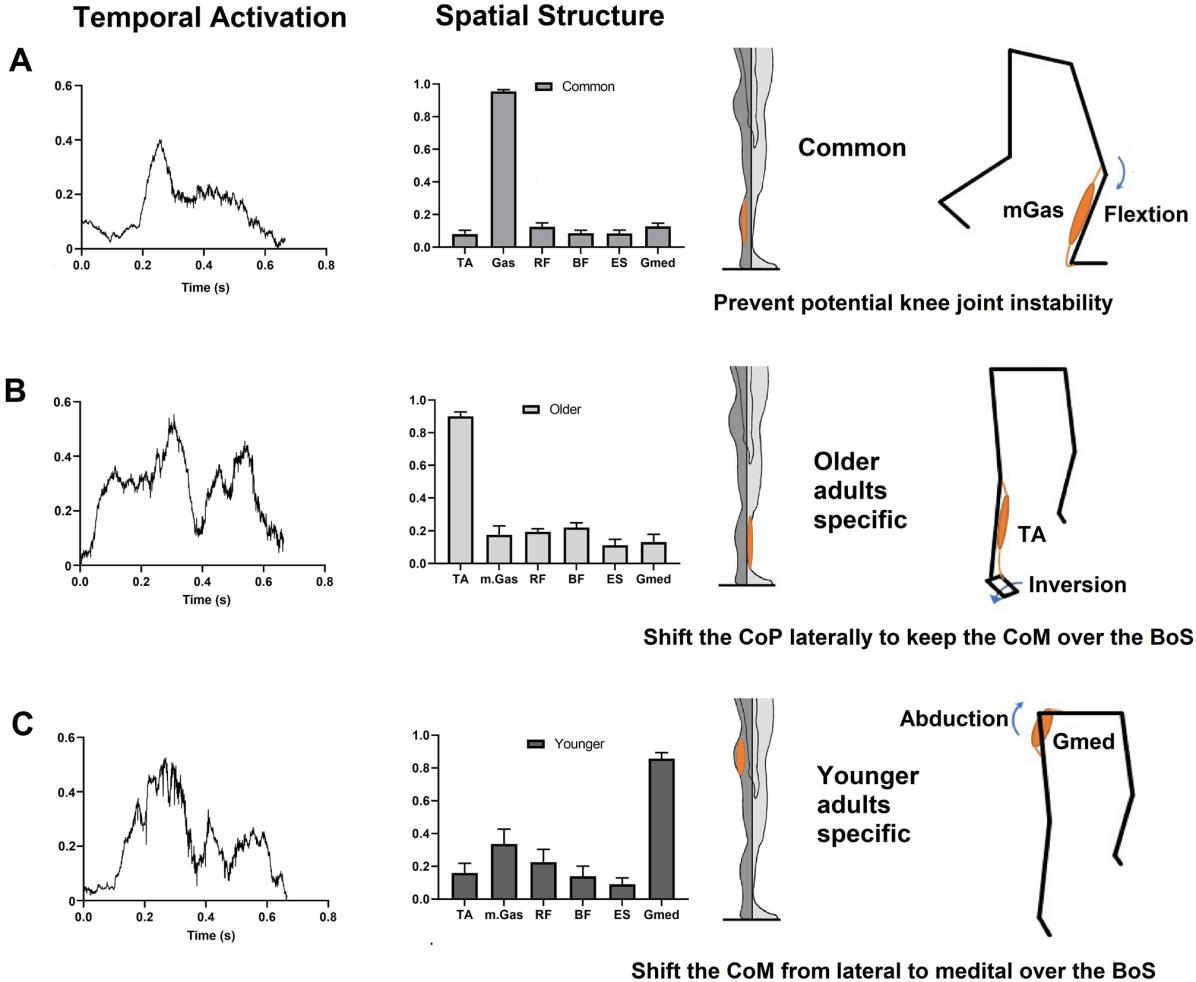
Common (top) and specific (middle: older, and bottom: younger) muscle synergy clusters. The left column displays the temporal recruitment coefficients. The middle column showcases the spatial component, indicating the relative contribution of each muscle. The right column highlights the predominant muscle within the spatial synergy component. (**A**) Common muscle synergy in both older and younger adults showed prominent activation of the mGas. (**B**) The older adults-specific muscle synergy was characterized by the prominent activation of the TA. (**C**) The younger adults-specific muscle synergy was characterized by the prominent activation of the Gmed. Abbreviations: mGas: medial gastrocnemius; TA: tibialis anterior; Gmed: gluteus medius; CoP: center of pressure; CoM: center of mass; BoS: base of support.

Muscle synergies of all participants were grouped into 4 clusters (see the supplementary material). Within these 4 clusters, we identified common and group-specific muscle synergies between older and younger adults. We observed a cluster showing good reliability (ICC value ≥ 0.75) that was consistent across all participants. This particular cluster exhibited predominant mGas muscle activation. Group-specific muscle synergies had excellent reliability (ICC value ≥ 0.90). The older adult specific muscle synergy cluster had predominant TA muscle activation, while younger adults specific muscle synergy cluster showed predominant Gmed activation (Fig. 7).

## Discussion

The purpose of this study was to investigate age-related changes in knee extensor eccentric control and muscle coordination patterns and the relationship of postural stability with knee extensor eccentric control while walking on a compliant (foam) surface with unpredictable properties (stiffness and damping). We found that knee extensor eccentric control during SLS was positively correlated with the balance control of protective stepping. Compared to younger adults, older adults demonstrated reduced knee extensor eccentric work, EMG burst duration, and showed prominent TA muscle activation (as opposed to prominent Gmed activation in younger adults) in muscle synergy during SLS of the perturbed leg. In addition, decreased swinging (trailing) leg protective step vertical velocity and MoS were observed in older adults.

### Relationship between knee extensor eccentric control and compensatory stepping stability

Loss of walking balance due to unexpected and/or partially expected ground balance challenges such as slip, trip, surface drops from uneven or compliant surfaces can be recovered by a protective stepping reaction^30–32^. A protective step lift-off during SLS, however, increases the body weight load on the perturbed leg and this requires additional negative eccentric force generation to stabilize the SLS phase of the perturbed leg.

Once protective stepping is initiated in response to a ground balance challenge, knee extensor eccentric control in the perturbed leg during SLS becomes critical as the initial defense against unrecoverable limb collapse^33^. Most of the negative work, which is needed to absorb the impact from ground perturbation, is created by the knee joint eccentric control, compared to control at the ankle and hip joints^34,35^. Therefore, sufficient knee extensor eccentric control is required to decelerate the body’s falling momentum, preventing the risk of limb collapse in the perturbed leg^36^ and provide postural stability in the SLS phase for the successful protective stepping^28^.

Indeed, our findings demonstrated a correlation between knee extensor eccentric work of the perturbed leg and balance control during the first protective step touchdown (swinging leg velocity and MoS). In other words, having better eccentric control at the knee joint during SLS leads to a more effective protective step to complete balance recovery. This indicates that insufficient eccentric control during SLS could result in incomplete and/or unstable balance recovery by the first protective step touchdown, and require additional protective steps and/or other types of recovery reactions (e.g., grasp or touch an object for support)^37,38^.

### Age-related differnces in knee extensor eccentric control and lower limb muscle coordination patterns

Lower extremity muscle strength decreases by 3–4% annually from age 70^39^. Force generation at the ankle, knee, and hip joints during dynamic balance reaction is highly associated with falls in older adults^40^. At the knee joint, the negative eccentric work reduces the risk of falls by absorbing falling momentum during single limb landing and slowing down the body’s forward momentum, thus preventing postural instability during SLS^38,41^.

We found that older adults have reduced knee extensor eccentric work and EMG burst duration compared to younger adults. This observation suggests that the deficiencies in eccentric control at the knee joint may be linked to heightened postural instability in older adults, particularly when they lack access to additional protective balance responses, such as reach-to-grasp movements. Indeed, force generation through knee extensor eccentric contraction is more variable than concentric contraction in older adults^42^, and the steadiness of knee extensor eccentric force generation distinguishes older fallers from older non-fallers^43^.

Age-related deterioration in lower extremity muscle strength and function leads to neuromuscular modification in older adults to regain standing balance during SLS^44^. The outcome of our muscle synergy analysis in this study showed that older adult-specific synergy involves highly activated TA muscle. During the initial phase of SLS, the CoM moves laterally toward the supporting (SLS) leg to prepare for swinging of the opposite leg, therefore, lateral stability is challenged in the frontal plane^45^. Ankle inversion torque generated by the TA muscle during single leg stance shifts the center of pressure (CoP) laterally^46^. Given that the CoP should be continuously moved toward the CoM to keep the CoM over the base of support for maintaining balance, the older adults-specific predominant TA activation appears to be one of the movement strategies for lateral balance control at the ankle joint level. At the hip joint level, however, the lateral balance can be more actively controlled by hip abduction when the CoM is shifted laterally. Indeed, the younger adults-specific muscle synergy showed greater activation of the Gmed. Considering reduced hip abduction torque-time production with age^47^, however, the ankle strategy is shown to be a neuromuscular modification for balance control in older adults. Our findings regarding the work at the ankle and hip joints also suggest that older adults tend to rely on ankle joint control to regain postural stability during the SLS phase, while younger adults more actively engage the hip joint. In the meantime, the activation of the medial gastrocnemius (mGas) was a prominent component of the observed muscle synergy in both older and younger adults. Given that the mGas is a biarticular muscle, with attachments at both the ankle and knee joints, the mGas contributes to generating compressive shear force at the knee joint when the foot is dorsiflexed during the SLS^48^. The compressive shear force created by the mGas stabilizes the SLS balance by preventing anterior tibial translation and attenuating the valgus loading at the knee joint^5,49^.

### Age-related differences in postural stability and its relationship to muscle activation patterns

We observed that older adults exhibited significantly greater postural sway in the A-P and M-L directions during the perturbed leg SLS phase. This indicates that even when the perturbed leg is not collapsed, insufficient knee extensor eccentric control results in a weakened and less stable perturbed leg, thereby increasing postural instability, which relates to potential fall risk. In addition, reduced MoS and swinging speed of the trailing leg protective step in older adults demonstrate that insufficient eccentric control during SLS leads to unstable and/or less effective balance recovery by the trailing leg protective (compensatory) step.

### Clinical significance

Our findings highlight the potential importance of knee extensor eccentric control for stabilizing the center of mass during walking balance responses to external ground perturbation. This provides evidence supporting future investigations into eccentric strength rehabilitation training for older adults and older frail adults (e.g., wall stand-to-sit on various challenging ground surfaces). In addition, the muscle synergy results further support the inclusion of hip abduction/adduction strengthening rehabilitation training for older adults as a means to improve lateral balance control, providing a more active movement strategy option. Nevertheless, it should be noted that previous studies have demonstrated the relevance of ankle moments for stabilizing the CoM^50,51^ and the aging-related redistribution of mechanical work from distal (the ankle joint) to proximal (the hip joint) during downhill walking are significantly smaller than during uphill walking^52^. These findings suggest that age-differences may be more prevalent in the ankle and knee, and less prevalent in the hip. Therefore, future comparative analyses of joint work at the ankle and hip should be conducted to better understand whether the hip and knee or knee and ankle or all three joints should be the focus of balance rehabilitation and fall prevention interventions.

### Limitations

There are a few limitations of the present study that should be acknowledged. Given our small sample size, the study’s results must be interpreted with caution. Future research with a larger sample size is necessary to assess the entire older population more accurately. In order to characterize muscle synergy patterns for balance recovery strategies, only the first response of each participant (a single trial) was analyzed as we were interested in capturing the naïve response during the first exposure to the balance challenge on the compliant surface. However, there can be a risk of bias in this method when evaluating a single trial from each subject. Specifically, computing NNMF in just one trial may result in the following limitations: 1) Generalizability (Representability): Results from a single trial can be susceptible to the influence of noise, variability, and atypical behaviors during the reaction motion, potentially rendering them unrepresentative of typical muscle activation patterns. To address within-subject variability in our method, we set the variability accounted for (VAF) threshold to 90, meaning that only the synergies explaining 90% of the data were used in the muscle synergy analysis. Between-subject variability exists in the spatial components of muscle synergies as well. Therefore, we used a statistical clustering method based on the intra-class correlation coefficient (ICC). Muscle synergies were categorized as common synergies if the ICC was greater than 0.75, age-specific synergies if the ICC was greater than 0.9. Through this approach, we mitigated the risk of capturing atypical motion and improved acquisition of representative measures. 2) Muscle Synergy Algorithm Stability: The NNMF algorithm is iterative and sensitive to the initial starting point of each iteration, which may lead to convergence in local minima. Therefore, the repeatability (stability) of NNMF outputs may not be guaranteed. To mitigate this risk and enhance stability, we used 20 random starting points (replicates) in each run of the algorithm. 3) Interpretation: Due to our small sample size, interpretating the muscle activation patterns we observed can be challenging when trying to conclude whether these age-specific muscle synergies apply to the entire population. Therefore, a larger sample size is required to improve the stability of muscle synergy interpretation and better support the identification of age-related movement strategies arising from the intrinsic variations in motor behavior.

Finally, to further validate our findings, future studies will involve the inclusion of additional muscles in the upper body and arms, as they contribute to overall postural balance control during the SLS phase. Additionally, it is important to incorporate faller populations in order to broaden the scope of our research.

## Conclusions

Knee extensor eccentric control during SLS was positively correlated with postural stability while walking on a compliant surface. Reduced knee extensor eccentric control in older adults was associated with increased postural sway and compromised swinging leg balance control in older adults. The muscle synergy analysis demonstrated that older adults exhibit a specific muscle synergy with highly activated TA muscle for postural control at the ankle joint level, while younger adults show prominent Gmed activation for postural control at the hip joint level during the SLS phase of balance response. These findings provide insights into age-related changes in neuromuscular control and movement strategies for balance control, supporting rehabilitation interventions for older adults that focus on improving knee extensor eccentric control and strengthening hip abduction/adduction.

### Supplementary Information

Supplementary Information.

## Data Availability

The datasets generated and analyzed during the current study are available from the corresponding author on reasonable request.
