# Supplementary material for: Age-related differences in lower limb muscle activation patterns and balance control strategies while walking over a compliant surface
Source: Sci Rep. 2023 Oct 2;13:16555. doi: 10.1038/s41598-023-43728-0 (PMC10545684; doi:10.1038/s41598-023-43728-0)
Supplement: Supplementary file 1 — Supplementary Information. [file 41598_2023_43728_MOESM1_ESM.pdf]

Article

Age-related differences in lower limb muscle activation patterns and balance control strategies while walking over a compliant surface

Woohyoung Jeon [1,*], Ahmed Ramadan [2], Jill Whitall [3], Nesreen Alissa [3], and Kelly Westlake [3,*]

1 Department of Health & Kinesiology, University of Texas at Tyler, TX, USA
2 Department of Biomedical Engineering, University of Minnesota Twin Cities, Minneapolis, MN, USA; aramadan@umn.edu
3 Department of Physical Therapy & Rehabilitation Science, University of Maryland School of Medicine, Baltimore, MD, USA; Nesreen.Alissa@som.umaryland.edu, JWhitall@som.umaryland.edu, KWestlake@som.umaryland.edu

* Correspondence: wjeon@uttyler.edu, kwestlake@som.umaryland.edu

Muscle synergies of all participants were grouped into 4 clusters, as shown below.

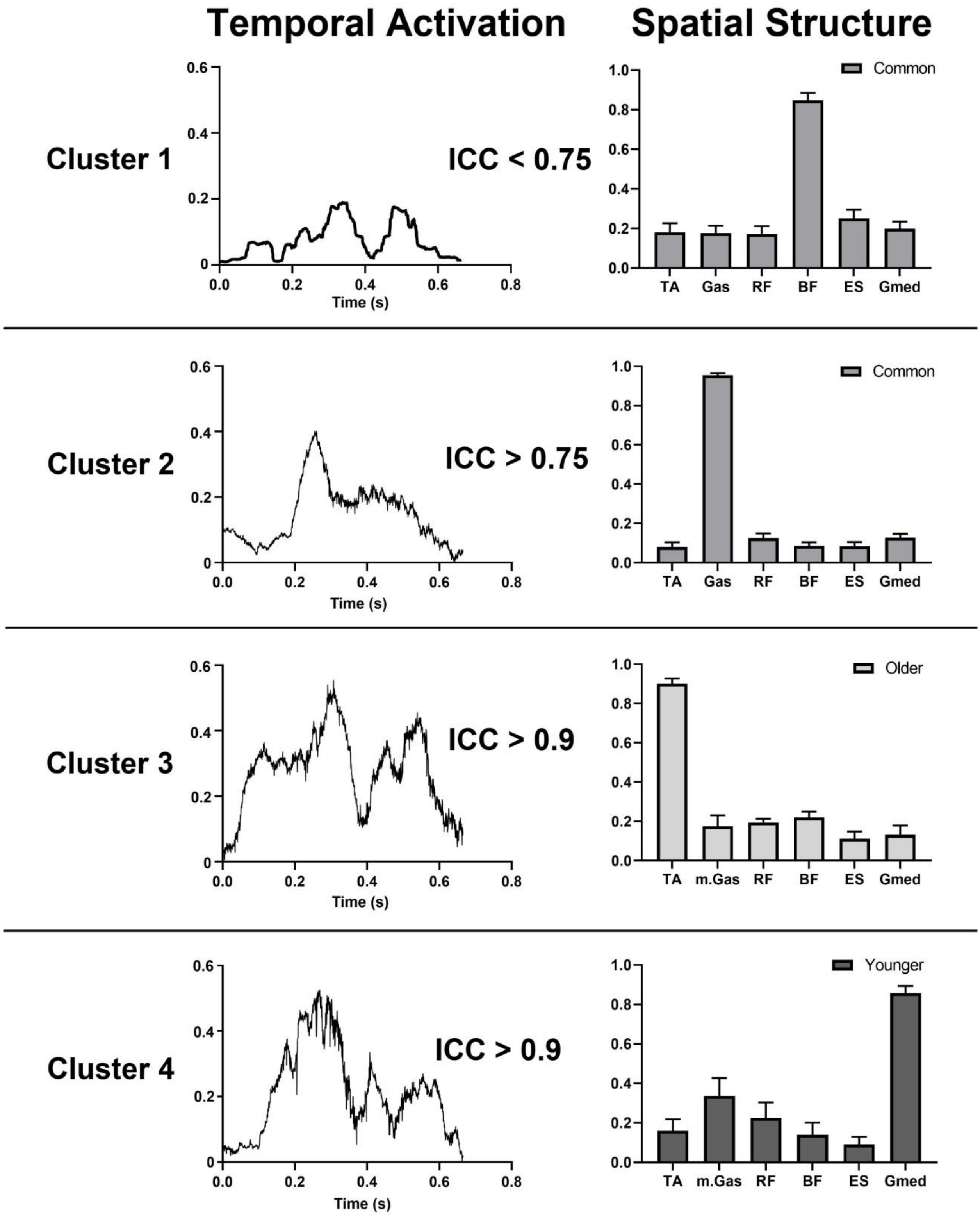

ICC = Intra-class Correlation Coefficient
